# Association of Telomere Length with Colorectal Cancer Risk and Prognosis: A Systematic Review and Meta-Analysis

**DOI:** 10.3390/cancers15041159

**Published:** 2023-02-11

**Authors:** Svenja Pauleck, Jennifer A. Sinnott, Yun-Ling Zheng, Shahinaz M. Gadalla, Richard Viskochil, Benjamin Haaland, Richard M. Cawthon, Albrecht Hoffmeister, Sheetal Hardikar

**Affiliations:** 1 Huntsman Cancer Institute, University of Utah, Salt Lake City, UT 84112, USA; 2 Medical Department II, Division of Gastroenterology, University of Leipzig Medical Center, 04103 Leipzig, Germany; 3 Department of Pediatrics, University of Utah, Salt Lake City, UT 84108, USA; 4 Department of Statistics, The Ohio State University, Columbus, OH 43210, USA; 5 Lombardi Comprehensive Cancer Center, Georgetown University Medical Center, Washington, DC 20057, USA; 6 Division of Cancer Epidemiology and Genetics, National Cancer Institute, Bethesda, MD 20892, USA; 7 Department of Population Health Sciences, University of Utah, Salt Lake City, UT 84108, USA; 8 Department of Human Genetics, University of Utah, Salt Lake City, UT 84108, USA; 9 Fred Hutchinson Cancer Research Center, Seattle, WA 98109, USA

**Keywords:** colorectal cancer, telomere length, risk, survival, meta-analysis

## Abstract

**Simple Summary:**

Colorectal cancer risk and survival have previously been associated with telomere length in peripheral blood leukocytes and tumor tissues. We quantitatively assessed these associations through a systematic review and meta-analysis. Following PRISMA guidelines, we identified relevant studies through database searches, and performed meta-analyses using random effects models. We found no association between telomere length in circulating leukocytes and the risk of developing colorectal cancer, however, shorter leukocyte telomeres were associated with a worse survival in patients with colorectal cancer. Therefore, telomere length may serve as a potential biomarker especially for colorectal cancer prognosis. Larger prospective cohort studies are needed to further confirm this potential association.

**Abstract:**

(1) Background: Colorectal cancer risk and survival have previously been associated with telomere length in peripheral blood leukocytes and tumor tissue. A systematic review and meta-analysis of the literature was conducted. The PubMed, Embase, and Web of Science databases were searched through March 2022. (2) Methods: Relevant studies were identified through database searching following PRISMA guidelines. Risk estimates were extracted from identified studies; meta-analyses were conducted using random effects models. (3) Results: Fourteen studies were identified (eight on risk; six on survival) through systematic review. While no association was observed between circulating leukocyte telomere length and the risk of colorectal cancer [overall OR (95% CI) = 1.01 (0.82–1.24)], a worse survival for those with shorter telomeres in leukocytes and longer telomeres in tumor tissues was observed [Quartile_1_/Quartile_2–4_ overall HR (95% CI) = 1.41 (0.26–7.59) and 0.82 (0.69–0.98), respectively]. (4) Conclusions: Although there was no association with colorectal cancer risk, a poorer survival was observed among those with shorter leukocyte telomere length. Future larger studies evaluating a potentially non-linear relationship between telomeres and colorectal cancer are needed.

## 1. Introduction

Telomeres are the nucleotide repetitive structures (TTAGGG) at the end of eukaryotic chromosomes that are sheltered by a protein complex [1,2]. Shortened by every DNA replication cycle, physiologically, the enzyme telomerase reverse transcriptase (TERT) maintains the telomere length in highly proliferative cells, such as stem cells [1,3]. In contrast, in most somatic cells, telomeres shorten with each cell division. Critically short telomeres activate the DNA damage response pathways and induce replicative senescence or apoptosis [4,5,6,7]. The amount of telomere attrition depends on genetic and environmental factors, including oxidative stress, genetic variation, and epigenetic changes such as histone modifications [8,9]. Cancer cells inhibit the process of apoptosis through dysfunction of the telomeric sheltering complex and generate extremely short telomeres [2]. These shortened and dysfunctional telomere structures form breakage-fusion bridge cycles that induce chromosomal instability [10,11], a hallmark of oncogenesis [12]. To achieve a high replicative potential, cancer cells activate the telomerase enzyme or the alternative lengthening of the telomeres (ALT) mechanism, consequently obtaining immortality [2,12,13,14,15]. Telomeres are crucial in tumorigenesis, and both shortening and lengthening of telomeres may promote tumor development and progression [16].

Several studies have suggested that shorter telomeres measured in circulating blood leukocytes are a risk factor for cancer development including colorectal cancer [16,17]. Two meta-analyses on the association between circulating leukocyte telomere length and colorectal cancer risk reported inconclusive results and are restricted by the limited number of included studies [18,19]. Two recent studies have reported a statistically significant association between colorectal cancer risk and circulating leukocyte telomere length [20,21]. However, these reports were not included in the two previously published meta-analyses.

Previous studies that looked at the association between telomere length and colorectal cancer survival have either utilized peripheral blood leukocytes for circulating telomere length measurement or have measured telomere length in preserved tumor tissues recovered during surgery. Two previously published meta-analyses suggested poorer colorectal cancer outcomes among patients with longer telomere length in tumor tissues and shorter telomere length in peripheral blood leukocytes [22,23]. However, these studies combined results from studies on tumor tissues and circulating leukocytes making them prone to biases.

The overall aim of this systematic review and meta-analysis was to evaluate telomere length measured in peripheral blood leukocytes as a potential predictive biomarker for colorectal cancer risk, as well as to assess the potential for telomere length measured in circulating leukocytes or tumor tissues to serve as a prognostic biomarker among patients with colorectal cancer.

## 2. Materials and Methods

### 2.1. Information Source and Search Strategy

This scoping review protocol was registered in Open Science Framework (OSF) online public database (registration DOI: https://doi.org/10.17605/OSF.IO/VRHJP (accessed on 20 January 2023)). The meta-analysis followed the ‘Preferred Reporting Items for Systematic Reviews and Meta-Analyses’ (PRISMA) guidelines [24], and the search was structured according to the PICOT strategy [25]. Using the PICOT strategy, the eligibility criteria were as follows: (P) individuals at risk for development of colorectal cancer (analysis for risk) and patients with a colorectal cancer diagnosis (analysis for survival), (I) observational studies for risk or survival after colorectal cancer, (C) comparing telomere length measured in peripheral blood leukocytes or tumor tissue with individuals’ risk or survival, (O) primary outcome measures were colorectal cancer risk and survival after colorectal cancer diagnosis, with (T) a follow-up of up to 20 years. We searched PubMed, Embase, and Web of Science databases through March 2022 for original research articles evaluating the role of telomere length in colorectal cancer risk and progression. We used the following Medical Subject Headings (MeSH) terms in PubMed: ’Colorectal Neoplasms’ AND ‘Telomere’ AND ‘Survival’ OR ‘Mortality’ OR ‘Death’ OR ‘Disease Progression’ OR ‘Prognosis’ OR ‘Risk’ OR ‘Risk Assessment’ OR ‘Probability’ OR ‘Odds Ratio’. The search was limited to human studies published in English language. We modified our search criteria slightly according to the database, as necessary. The search terms used for each database are listed in Supplementary Table S1.

### 2.2. Study Selection

Studies were considered as eligible for the analysis on colorectal cancer risk if (i) they investigated the association of telomere length in circulating leukocytes with the risk of colorectal cancer; and (ii) risk analyses were reported as odds ratios (ORs) or relative risks (RRs). For the survival analysis, we selected studies that (i) measured telomere length in bowel tissue retrieved from colorectal cancer surgery or circulating leukocytes, and evaluated associations with overall or colorectal cancer specific survival; and (ii) reported their results as 5-year survival rates, hazard ratios (HRs), or relative risks (RRs).

### 2.3. Data Extraction

**Figure 1:**
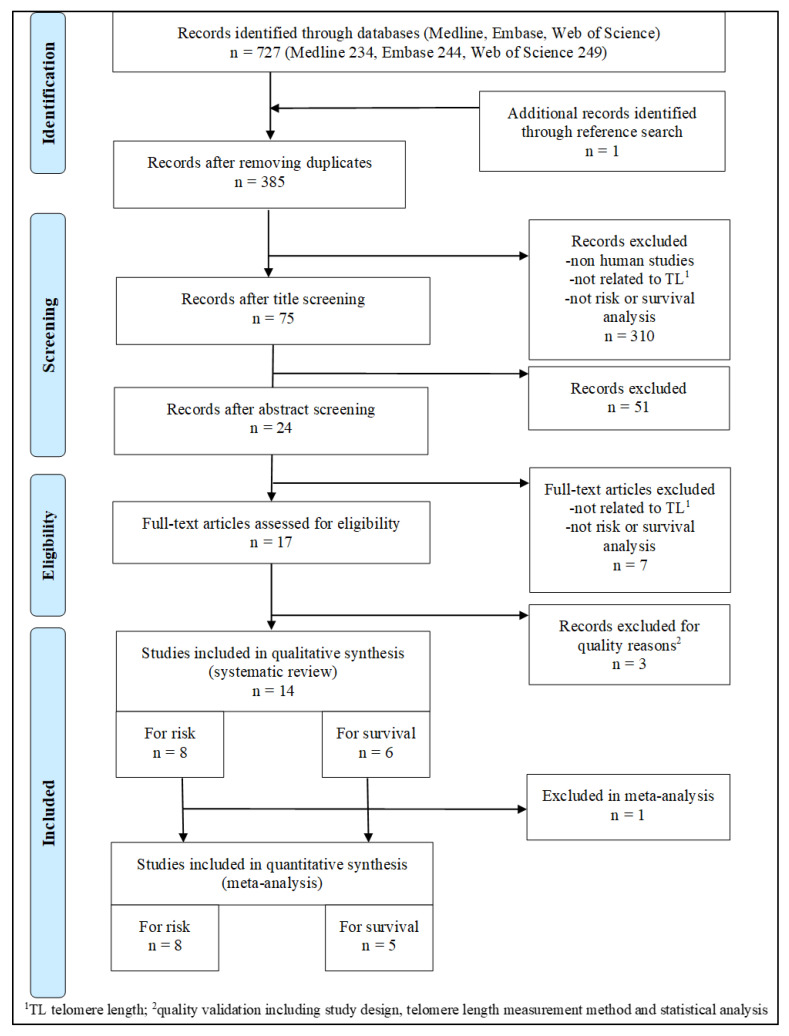
PRISMA flow diagram showing the selection and filtering steps for eligible studies for the association of telomere length in leukocytes or tumor tissue with colorectal cancer risk and survival.

Database searching and data extraction for the selected articles was performed by a single abstractor (S.P.) after training by a librarian specializing in literature synthesis at the University of Utah Health Sciences Library. Any discrepancy or controversial eligibility for article inclusion was discussed with S.H and J.A.S. After running the database searches, results were filtered for duplicates, titles, and abstracts; relevant full text articles were retained (Figure 1).

The selected articles were studied in detail and relevant data was extracted including author, publication year, manuscript title, journal, study design, study participant details (age, sex, and country), type of biospecimen used for telomere measurement, method of telomere length measurement, type of analysis (risk or survival), and ORs or HRs adjusted for the greatest number of covariates (i.e., the most adjusted model).

### 2.4. Data Synthesis and Statistical Analysis

We conducted separate meta-analyses for studies of colorectal cancer risk and survival using random effects meta-analysis models. For the risk analysis, ORs and 95 % confidence intervals (CI) were extracted from included articles. Assuming a Gaussian distribution of log transformed telomere length, we transformed all ORs to compare Q4 (longest quartile) vs. Q1 (shortest quartile) of telomere length (Material S1). For the survival analysis, we extracted all HRs and converted relative ratio results to HRs assuming a normal distribution for telomere length. HRs were aligned to make study results comparable as Q1 (shortest quartile) vs. Q2–Q4 (longer quartiles) of telomere length (Material S1). Using a random effects model, we meta-analyzed results from included studies to compute overall OR and HR for the association of telomere length with colorectal cancer risk and survival, respectively. Inter-study heterogeneity was assessed using Cochrane’s Q test, I^2^ statistic, and τ^2^ statistic, and was classified as low, moderate, or substantial [26]. Prediction intervals were calculated for meta-analyses with more than two studies included. The influence of included studies on the overall random effects model were explored by dropping one study at a time and observing the change in the overall risk estimates. Potential publication bias was assessed through funnel plot asymmetry for meta-analyses where more than three studies were available. All calculations were conducted in R 4.0.2 (R Core Team 2020), using the *meta* [27] and *dmetar* [26] packages. Figures were constructed using the *ggplot2* [28] package.

## 3. Results

### 3.1. Study Selection

In total, 727 research articles were identified through our database search: 234 in PubMed, 244 in Embase, and 249 in Web of Science (Figure 1).

One study was identified through cross-reference search. After removing duplicates, 385 articles were eligible for further consideration. After screening for relevant titles and abstracts, 24 articles met our eligibility criteria. We further excluded seven articles after full-text review as they were not related to the research question of interest. We also excluded three articles after assessing for quality as they lacked key information on study design, telomere length measurement, or statistical analyses, including adjustment for key confounders and proper statistical modeling techniques (Figure 1). Ultimately, eight studies on colorectal cancer risk and six studies on survival were included in our systematic review. For the meta-analyses, we further excluded one study on survival analysis as it did not provide any risk estimates (only 5-year survival rates were included as results).

### 3.2. Study Characteristics and Findings

The general characteristics of the included studies are summarized in Table 1. They summarize the study details for colorectal cancer risk (Table 2), and survival (Table 3).

Of the three retrospective [20,29,30] and five prospective [21,31,32,33,34] studies evaluating the association between telomere length and colorectal cancer risk, three studies each were conducted in the USA [29,31,32] and China [20,21,34], while two were from the UK [30,33]. Participants’ age ranged from 21 to 89 years. One study stratified by age groups (≤50 years vs. >50 years) [29]. We only included results for the older age group for this study to align participant characteristics with the other studies included in the meta-analysis. Varying DNA extraction methods were utilized by the studies, including assays from QIAgen systems [21,30,31,32,34], phenol/chloroform [29], or RelaxGene [20] systems. All studies quantified telomeres by a quantitative PCR method adjusted at least for age and sex.

Among the studies evaluating the role of telomeres in colorectal cancer survival, five were conducted in Europe or Australia [35,36,37,38,39] and one in China [40]. The age of the participants ranged from 26 to 96 years. DNA was extracted by column-based systems by QIAgen systems [35,36,38] or an electrolyte-based system by TIANGEN [40]. Telomere length in circulating leukocytes was measured by unified quantitative PCR [35,40], whereas telomere length in bowel tissue was measured using Southern Blot [36,37] or multiplex quantitative PCR by calculating the ratio of telomere length in tumor tissue to adjacent healthy mucosa [38,39]. One study only presented 5-year survival rates (rather than HR) and was therefore not included in our meta-analysis [39].

### 3.3. Meta-Analysis

**Figure 2:**
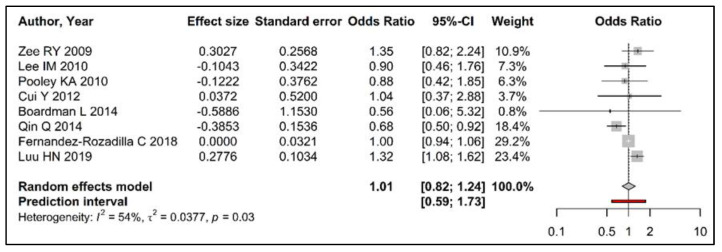
Forest plot summarizing the association between telomere length (comparing longest quartile vs. shortest) in peripheral blood leukocytes and risk of colorectal cancer using random effects model. (References: Zee RY 2009 [32], Lee IM 2010 [31], Pooley KA 2010 [33], Cui Y 2012 [34], Boardman L 2014 [29], Qin Q 2014 [20], Fernandez-Rozadilla C 2018 [30], Luu HN 2019 [21]).

The overall OR comparing the longest quartile (Q4) with the shortest quartile (Q1) did not suggest any association between telomere length in circulating leukocytes and colorectal cancer risk (OR [95 % CI] 1.01 [0.82–1.24]) (Figure 2).

**Figure 3:**
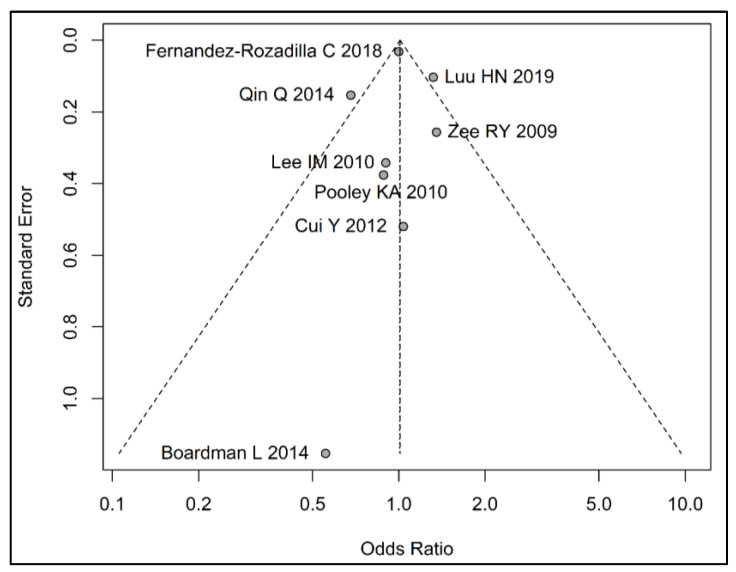
Funnel plot assessing potential publication bias of included studies (n = 8) in meta-analysis of telomere length (comparing longest quartile vs. shortest) and colorectal cancer risk. (References: Zee RY 2009 [32], Lee IM 2010 [31], Pooley KA 2010 [33], Cui Y 2012 [34], Boardman L 2014 [29], Qin Q 2014 [20], Fernandez-Rozadilla C 2018 [30], Luu HN 2019 [21]).

Similar results were observed for the comparisons of quartiles 2 and 3 with Q1 (Supplementary Figure S1). We observed a moderate heterogeneity in our random effects model comparing Q4 to Q1 with Cochrane’s Q = 15.22 (*p* = 0.03), I^2^ statistic = 54% and τ^2^ statistic = 0.03, and a wide prediction interval of 0.59 to 1.73 (Figure 2). When dropping one study at a time and recalculating overall estimates, we did not identify any study that influenced our overall findings significantly. The visual inspection of the funnel plots did not show any evidence of potential publication bias (Figure 3, Supplementary Figure S2).

We did not observe any significant heterogeneity by study design (retrospective vs. prospective studies) (OR [95% CI] = 1.22 [0.97–1.53] and 0.86 [0.64–1.15] for prospective vs. retrospective studies, respectively; pheterogeneity = 0.06).

**Figure 4:**
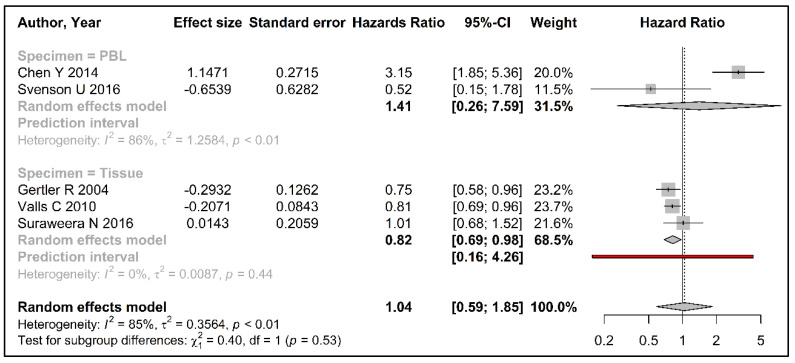
Forest plot summarizing the association between telomere length (comparing shortest quartile vs. longer quartiles) and survival of colorectal cancer within subgroups of peripheral blood leukocytes and tumor tissue using random effects model. (References: Chen Y 2014 [40], Svenson U 2016 [35], Gertler R 2004 [36], Valls C 2010 [37], Suraweera N 2016 [38]).

The association between telomere length and colorectal cancer survival is summarized in Figure 4.

We observed contrasting associations with colorectal cancer survival for leukocyte vs. tissue telomere length in our meta-analyses; shorter telomeres in circulating leukocytes were associated with a worse survival (HR [95% CI] 1.41 [0.26–7.59]), although this was not statistically significant, while shorter telomeres in tissues were associated with a better survival after colorectal cancer diagnosis (HR [95% CI] 0.82 [0.69–0.98]) (Figure 4). We were unable to assess publication bias through heterogeneity testing or funnel plots due to the limited number of studies. The prediction interval for survival analysis with telomere length measured in tumor tissues showed a wide range from 0.16 to 4.26.

## 4. Discussion

Our results did not suggest an association between leukocyte telomere length and colorectal cancer risk after meta-analysis of eight studies evaluating colorectal cancer risk. In this meta-analysis of five studies on telomere length with colorectal cancer survival, we observed a worse overall survival with shorter telomeres in circulating leukocytes and longer telomeres in tumor tissue. No evidence of publication bias was observed, though we observed some heterogeneity in previously published studies.

To the current meta-analysis, we were able to add two recent studies on telomere length and colorectal cancer risk [21,30] that were not included in the previously published meta-analyses [18,19,41]. A previous systematic review and meta-analysis of seven studies by Naing et al. published in 2017 examined telomere length in circulating leukocytes and its association with colorectal cancer risk [18]. They concluded that there was overall no association between telomere length and risk of colorectal cancer and a suggestive association for retrospective studies only, reporting wide heterogeneity among included studies [18]. We did not observe a difference between prospective or retrospective study designs in our analysis. Two other meta-analyses that included all types of cancer also did not observe an association of leukocyte telomere length with risk of colorectal cancer [19,41]. Both these meta-analyses were limited by the number of studies reporting results on colorectal cancer; these meta-analyses included three [19] and two studies [41], respectively, on colorectal cancer risk. We compared our reparametrized results (telomere length in quartiles) with these three meta-analyses shown in Supplementary Table S2 [18,19,41]. Differences were observed for the studies by Cui et al. 2012 and Boardman et al. 2017. Both these studies reported a non-linear association between telomere length and colorectal cancer risk, suggesting the importance of accounting for a non-linear association between telomere length and colorectal cancer risk. In fact, this might be a reason for an inconclusive association between telomere length and colorectal cancer risk in previously published studies [30,31,32].

Consistent with two previously published meta-analyses from 2016 and 2017, we reported that longer telomeres in tumor tissues and shorter telomeres in leukocytes were associated with a worse overall survival after colorectal cancer [22,23]. The meta-analysis from 2016 is limited in its reporting of the association of telomere length with colorectal cancer survival as it included only one study on colorectal cancer survival [23]. The more recent meta-analysis from 2017 reported results on overall and disease-/progression-free survival with a suggestive association for overall survival and no association for disease-free survival [22]. This analysis reported combined results for both leukocyte and tumor tissue telomere length [22], however, this may be complicated by contrasting associations between leukocyte and tissue telomeres with colorectal cancer survival and the cancelation of opposing effects in overall meta-analyses. In the current study, we present separate overall estimates for leukocyte and tissue telomere length with survival. The inverse association between studies evaluating telomere length in circulating leukocytes vs. tissues might be due to differences in telomere length measurement. Two out of three studies that evaluated telomere length in tissues used Southern Blot for telomere analysis vs. all the studies in circulating leukocytes that used a PCR-based method (Table 3). Although previous studies suggest that telomeres in leukocytes as well as malignant adenomatous tissues tend to be shorter than non-cancerous polyps [42], it is likely that telomeres differ between cell types according to their mitotic potential [43]. A recent study by Demanelis et al. 2020, analyzed telomere lengths in various tissue types [44]. They concluded that leukocytes possessed the shortest telomeres but were a good proxy for most tissues [44]. Future larger studies are needed to explore this association further.

Comprehensive studies on telomere-related gene expression in colorectal cancer have reported extreme shortening of telomere length in early-stage colorectal cancer that is compensated by the overexpression of telomere maintenance mechanisms [45,46]. Over time, telomere shortening seems to overcome this compensation leading to shorter telomeres in advanced colorectal cancer stages compared with earlier stage colorectal cancer [46]. Telomere length is a dynamic measure and continues to change over the cancer continuum (from cancer development to progression) owing to the contrasting effects of rapidly dividing cells with telomere attrition against telomere maintenance [45,46]. Telomere length in peripheral blood leukocytes seems to be shorter in cancer-free controls for up to 8 to 14 years pre-diagnosis, but telomere attrition decelerates closer to cancer diagnosis [47]. These findings suggest the need for the longitudinal measurements of telomere length in peripheral blood leukocytes in both risk and survival analyses. This meta-analysis included studies with telomere measurement at one time-point only that might not reflect sufficiently the correlation between telomere length and colorectal cancer development and progression over time.

Different DNA extraction methods (column-, phenol/chloroform-, or buffer system-based) were reported in the studies included in this meta-analysis. This may have contributed to some of the heterogeneity observed between studies. It has been previously reported that telomere lengths on DNA extracted through column-based systems are shorter compared with telomere lengths measured on DNA extracted using other systems [48]. Similar to our results, Zhang et al. observed a significant association with colorectal cancer risk by including studies with a precise DNA extraction method description and telomere length measurement by multiplex quantitative PCR [41].

Our study is limited by the small number of available studies evaluating this research question, the differences in DNA extraction and telomere length measurement methods in these studies, as well as a lack of consistency in reporting results for telomere length measurement. This observation is consistent with a recent report by Lindrose et al. 2021, that concluded that there is a lack of rigorous reporting of telomere measurement procedures in the published literature [49]. We recommend that future studies should report on telomere measurement methodology including DNA extraction and processing methods, PCR assays and, analytical approaches, particularly details on telomere length parametrization [49]. Additionally, the reporting on reproducibility and repeatability of telomere measurement methods is essential to allow for a better assessment of the published literature with an overall goal of improving the methodological quality of telomere-related studies [49].

Our meta-analysis includes recently published studies that have not been included in previous systematic reviews or meta-analyses [21,30,39]. Additionally, in contrast to previously reported meta-analyses, we have accounted for variability in statistical analyses of the included studies in our overall estimates through a reparameterization of telomere length, and aligned results from all included studies to compare the shortest quartile of telomere length with other quartiles. For future epidemiologic studies, we recommend that researchers consider a potential non-linear distribution for the association of telomere length with risk or survival of colorectal cancer. Furthermore, the recommendations by the Telomere Research Network and broader scientific community should be followed in order to guarantee a high quality of telomere-related research, and reduce potential errors [49,50].

## 5. Conclusions

We observed no association between telomere length in circulating leukocytes and the risk of developing colorectal cancer. We observed a possible association of shorter telomeres in circulating leukocytes, although it was not statistically significant, and of longer telomeres in tumor tissues with survival after colorectal cancer diagnosis. Thus, telomere length in circulating leukocytes and tumor tissues may have a potential for being prognostic biomarkers for colorectal cancer survival and may aid clinicians to identify patients that have a higher risk for adverse clinical outcomes. Heterogeneity among the included studies was observed, likely due to the limited number of included studies, differences in DNA extraction and telomere measurement methods, as well as a lack of the standard reporting of telomere parametrization. Future studies evaluating the relationship of telomeres with colorectal cancer risk and survival should follow the guidelines for telomere length measurement and reporting, to evaluate the potential of telomeres as predictive and prognostic biomarkers for colorectal cancer.

## Figures and Tables

**Table 1:** General characteristics of studies (n = 14) included in the meta-analysis of the association between telomere length and (a) colorectal cancer risk, and (b) colorectal cancer survival.

Author Year	Country	Study Design	Participants’ Characteristics (Cases/Controls)				
			n	Age	% Male	BMI	% Ever Smokers
(**a**) Risk analyses							
Zee RY 2009 [32]	USA	Prospective Case control	191/306	58.1 (±8.0)/60.5 (±8.7)	100	24.8 (±2.6)/25.2 (±2.9)	60.8/64.4
Lee IM 2010 [31]	USA	Prospective Case control	134/357	60.1 (±8.7)/60.7 (±8.6)	0	26.2 (±5.6)/25.9 (±4.9)	53.0/47.1
Pooley KA 2010 [33]	UK	Prospective Case control	185/406	64 (40–80)/64 (41–80)	nr ^1^	26.8 (±4.2)/26.3 (±4.0)	50/50
Cui Y 2012 [34]	China	Prospective Case control	441/549	58.5 (±8.7)/58.6 (±8.6)	0	24.6 (±3.3)/24.8 (±3.5)	2.5/3.8
Boardman L 2014 [29]	USA	Retrospective Case control	598/2212	48.3 (±8.3)/56.8 (±12.1)	50/52	27.6 (±6.1)/28.0 (±5.7)	52/49
Qin Q 2014 [20]	China	Retrospective Case control	628/1256	58.8 (±11.8)/58.8 (±11.4)	54.1/54.9	23.2 (±3.3)/23.0 (±3.2)	38.2/28.7
Fernandez-Rozadilla C 2018 [30]	UK	Retrospective Case control	211/106	66 (±8.8)/53 (±16.9)	53.08/48.11	nr ^1^	nr ^1^
Luu HN 2019 [21]	China	Prospective Case control	776/25,764	65.9 (±7.9)/62.72 (±7.6)	55.15/45.82	23.3 (±3.4)/23.3(±3.5)	39.6/31.8
(**b**) Survival analyses							
Gertler R 2004 [36]	Germany	Prospective overall survival	57	64.6 (±13.6)	52.6	nr ^1^	nr ^1^
Valls C 2011 [37]	Spain	Prospective overall survival	147	age (≤70) 46%	54.0	nr ^1^	nr ^1^
Chen Y 2014 [40]	China	Prospective overall survival	571	58.4 (±12.3)	54.7	nr ^1^	nr ^1^
Svenson U 2016 [35]	Sweden	Prospective CRC specific survival	130	70 (26–93)	52.31	nr ^1^	nr ^1^
Suraweera N 2016 [38]	UK, Australia	Prospective overall survival	281	nr ^1^	52.0	27.6 (±5.0)	47.8
Kroupa M 2019 [39]	Czech Republic	Prospective overall survival	661	68 (33–96)	62.8	nr ^1^	43.5

^1^ not reported.

**Table 2:** Characteristics of telomere length measurements in peripheral blood leukocytes of n = 8 studies and the reported colorectal cancer risk estimates compared with the calculated risk estimates (4th quartile vs. 1st quartile [Q4/Q1]).

Author Year	DNA Extraction Method	TL ^1^ Measurement Method	TL ^1^ Parametrization	Reported Risk Estimates (95%CI)	Calculated Risk Estimates (95% CI)
Zee RY 2009 [32]	QIAprep ^2^	RTL ^3^	Continuous	1.25 (0.86–1.81)	1.35 (0.82–2.24)
Lee IM 2010 [31]	QIAprep ^2^	RTL ^3^	Continuous	0.94 (0.65–1.38)	0.90 (0.46–1.76)
Pooley KA 2010 [33]	nr ^4^	RTL ^3^	TL ^1^ Q4 (shortest)/Q1 (longest)	1.13 (0.54–2.36)	0.89 (0.42–1.85)
Cui Y 2012 [34]	QIAamp ^2^	RTL ^3^	TL ^1^ Q1 (shortest)/Q3 TL ^1^ Q5 (longest)/Q3	1.56 (0.92–2.64) 1.61(0.94–2.75)	1.04 (0.38–2.88)
Boardman L 2014 [29]	phenol/chloroform	RTL ^3^	P10 (shorter)/P50	1.91 (1.07–3.41)	0.56 (0.06–5.32)
Qin Q 2014 [20]	RelaxGene ^5^	RTL ^3^	TL ^1^ Q1 (shortest)/Q4 (longest)	1.47 (1.09–1.99)	0.68 (0.50–0.92)
Fernandez-Rozadilla C 2018 [30]	QIAamp ^2^	RTL ^3^	Continuous	1.00 (0.88–1.14)	1.00 (0.94–1.07)
Luu HN 2019 [21]	QIAamp ^2^	RTL ^3^	TL ^1^ Q4 (longest)/Q1 (shortest)	1.32 (1.08–1.62)	1.32 (1.08–1.62)

^1^ TL telomere length; ^2^ by Qiagen; ^3^ RTL Relative telomere length (T/S ratio) by unified quantitative PCR; ^4^ not reported; ^5^ by TIANGEN.

**Table 3:** Characteristics of telomere length measurements in peripheral blood leukocytes or tumor tissue of n = 6 studies and the reported survival estimates compared with the calculated estimates (1st quartile vs. the longer quartiles [Q1/Q2–Q4]).

Author Year	Specimen	DNA Extraction Method	TL ^1^ Measurement Method	Follow-Up Time (months)	Survival Comparison Groups (%)	KM 5 yrs ^2^ Survival (%)	Reported Risk Estimates (95%CI)	Calculated Risk Estimates (95% CI)
Gertler R 2004 [36]	Tissue	QIAamp ^3^	TRF ^4^	75.5 (52–87)	TRF ratio > 0.9 (25) TRF ratio ≤ 0.9 (75)	25.6 78.2	3.30 (1.20–9.00)	0.75 (0.58–0.96)
Valls C 2011 [37]	Tissue	nr ^5^	TRF ^4^	45.1 (1.6–59.8)	TRF ratio > 1 (23.2) TRF ratio ≤ 1 (76.8)	55.2 64.6	2.44 (1.20–4.98)	0.81 (0.69–0.96)
Chen Y 2014 [40]	PBL ^6^	RelaxGene ^7^	RTL ^8^	28 (6–60)	RTL ≤ 0.704 (59.2) RTL > 0.704 (40.8)	52.6 70.3	2.43 (1.53–3.45)	3.15 (1.85–5.36)
Svenson U 2016 [35]	PBL ^6^	QIAamp ^3^	RTL ^8^	202	Q1 RTL (shortest) Q2–Q4 RTL	96.0 74.0	0.52 (0.15–1.76)	0.52 (0.15–1.76)
Suraweera N 2016 [38]	Tissue	DNAeasy ^9^	RTL ^8^	45.2	RTL continuous	nr ^5^	0.99 (0.75–1.32)	1.01 (0.68–1.52)
Kroupa M 2019 [39]	Tissue	DNAeasy ^9^	RTL ^8^	nr ^5^	RTL ratio < 0.9 RTL ratio ≥ 0.9	69.4 59.5	nr ^5^	None

^1^ TL telomere length; ^2^ Kaplan Meier 5 years survival time; ^3^ by Qiagen; ^4^ telomere restriction fragments (kb) by luminescence; ^5^ not reported; ^6^ peripheral blood leukocytes; ^7^ by TIANGEN; ^8^ relative telomere length (T/S ratio) by unified quantitative PCR; ^9^ DNeasy blood and tissue kit by Qiagen.

## Supplementary Materials

The following supporting information can be downloaded at: https://www.mdpi.com/article/10.3390/cancers15041159/s1, Table S1: Search terms for literature search within the three databases to evaluate the association of telomere length with colorectal cancer risk and survival; Material S1: Reparameterization details of odds ratios and hazard ratios from included articles; Figure S1: Forest plot summarizing the association between telomere length in peripheral blood leukocytes and risk of colorectal cancer using random effects model for quartiles of telomere length (a) quartile 2 vs. quartile 1; and (b) quartile 3 vs. quartile 1; Figure S2: Funnel plots assessing potential publication bias of included studies in meta-analysis of telomere length and colorectal cancer risk for quartiles of telomere length (a) quartile 2 vs. quartile 1; and (b) quartile 3 vs. quartile 1; Table S2: Comparison of our reparametrized odds ratios for the association between telomere length and colorectal cancer risk with three other published meta-analyses (Naing et al., 2017; Zhu et al., 2016; Zhang et al., 2017).
