# Supplementary material for: Association of Telomere Length with Colorectal Cancer Risk and Prognosis: A Systematic Review and Meta-Analysis
Source: Cancers (Basel). 2023 Feb 11;15(4):1159. doi: 10.3390/cancers15041159 (PMC9954736; doi:10.3390/cancers15041159)
Supplement: Supplementary file 1 [file cancers-15-01159-s001.zip › cancers-2140088-supplementary - XML/cancers-2140088-Supplementary.docx]

Supplementary Materials

Association of Telomere Length with Colorectal Cancer Risk and Prognosis: A Systematic Review and Meta-Analysis

Svenja Pauleck, Jennifer A. Sinnott, Yun-Ling Zheng, Shahinaz M. Gadalla, Richard Viskochil, Ben Haaland, Richard M. Cawthon, Albrecht Hoffmeister, Sheetal Hardikar

**Table S1.** Search terms for literature search within the three databases to evaluate the association of telomere length with colorectal cancer risk and survival.

| **Database** | **Search terms** |
| --- | --- |
| Medline | (("Colorectal Neoplasms"[Mesh] OR "colorectal cancer"[tiab] OR "colorectal neoplasms"[tiab] OR "colorectal carcinoma"[tiab] OR "colorectal tumor"[tiab] OR "colon neoplasm"[tiab] OR "cancer of colon"[tiab] OR "colon cancer"[tiab] OR "colon tumor"[tiab] OR "rectal neoplasm"[tiab] OR "cancer of rectum"[tiab] OR "rectal cancer"[tiab] OR "rectal tumor"[tiab]) AND ("Telomere"[Mesh] OR telomer[tiab] OR telomer's[tiab] OR telomera[tiab] OR telomeras[tiab] OR telomerasa[tiab] OR telomerase[tiab] OR telomerase's[tiab] OR telomeraseactivity[tiab] OR telomeraseassociated[tiab] OR telomerasedeficient[tiab] OR telomerasenull[tiab] OR telomerasepositive[tiab] OR telomerases[tiab] OR telomeraseses[tiab] OR telomerasespecific[tiab] OR telomerasic[tiab] OR telomerasing[tiab] OR telomerasing'[tiab] OR telomerator[tiab] OR telomere[tiab] OR telomere'[tiab] OR telomere's[tiab] OR telomerease[tiab] OR telomerebinding[tiab] OR telomerecat[tiab] OR telomered[tiab] OR telomerehunter[tiab] OR telomereindependent[tiab] OR telomereless[tiab] OR telomerelike[tiab] OR telomerenickel[tiab] OR telomereopathies[tiab] OR telomereres[tiab] OR telomeres[tiab] OR telomeres'[tiab] OR telomeresand[tiab] OR telomerespecific[tiab] OR telomereto[tiab] OR telomeretrimming[tiab] OR telomeric[tiab] OR telomericaggregates[tiab] OR telomerically[tiab] OR telomerics[tiab] OR telomerina[tiab] OR telomeriques[tiab] OR telomerisation[tiab] OR telomerisation'[tiab] OR telomerised[tiab] OR telomerization[tiab] OR telomerizations[tiab] OR telomerized[tiab] OR telomerless[tiab] OR telomermis[tiab] OR telomeropathies[tiab] OR telomeropathy[tiab] OR telomers[tiab] OR telomers'[tiab] OR telomerse[tiab] OR telomersyn[tiab]) AND ("Survival"[Mesh] OR "Mortality"[Mesh] OR "Death"[Mesh] OR "Disease Progression"[Mesh] OR "Prognosis"[Mesh] OR death[tiab] OR survival[tiab] OR mortality[tiab] OR progression[tiab] OR prognoses[tiab] OR "prognostic factors"[tiab] OR severity[tiab] OR risk[Mesh] OR risk[tiab] OR "Relative Risk"[tiab] OR "relative risks"[tiab] OR "risk assessment"[Mesh] OR "risk assessments"[tiab] OR "risk assessment"[tiab] OR "Health Risk Assessment"[tiab] OR probability[Mesh] OR probability[tiab] OR "odds ratio"[Mesh] OR "odds ratio"[tiab] OR "risk ratio"[tiab] OR "relative odds"[tiab] OR "risk ratios"[tiab] OR "odds ratios"[tiab] OR "relative odds"[tiab])) NOT Review[ptyp] AND "humans"[MeSH Terms] |
| Embase | ('colorectal cancer'/exp OR 'colorectal cancer' OR 'colorectal tumor'/exp OR 'colorectal tumor' OR 'colon tumor'/exp OR 'colon tumor' OR 'colon cancer'/exp OR 'colon cancer' OR 'rectum cancer'/exp OR 'rectum cancer' OR 'rectum tumor'/exp OR 'rectum tumor') AND ('telomere'/exp OR 'telomere' OR 'telomerase' OR 'telomerase'/exp OR telomerase OR 'telomere length'/exp OR 'telomere length' OR 'telomerase reverse transcriptase'/exp OR 'telomerase reverse transcriptase' OR 'telomere homeostasis'/exp OR 'telomere homeostasis') AND ('survival' OR 'survival'/exp OR survival OR 'survival rate'/exp OR 'survival rate' OR 'death' OR 'death'/exp OR death OR 'mortality' OR 'mortality'/exp OR mortality OR 'mortality rate'/exp OR 'mortality rate' OR 'mortality risk'/exp OR 'mortality risk' OR 'prognosis' OR 'prognosis'/exp OR prognosis OR 'progression free survival'/exp OR 'progression free survival') AND ([article]/lim OR [article in press]/lim OR [conference abstract]/lim OR [conference paper]/lim) AND ([article]/lim OR [article in press]/lim OR [conference paper]/lim OR [data papers]/lim) NOT [medline]/lim AND ('risk'/exp OR risk OR 'risk assessment'/exp OR 'risk assessment' OR 'risk factor'/exp OR 'risk factor' OR 'odds ratio'/exp OR 'odds ratio' OR 'probability'/exp OR probability) |
| Web of Science | TOPIC: ("colorectal cancer" OR "colorectal tumor" OR "colorectal neoplasm" OR "colon cancer" OR "colon tumor" OR "colon neoplasm" OR "rectum cancer" OR "rectum tumor" OR "rectum neoplasm")  Refined By:TOPIC: (telomere OR telomerase OR telomerase OR telomerase OR "telomere length" OR "telomerase reverse transcriptase" OR "telomere homeostasis") AND TOPIC: (risk OR odds OR survival OR "survival rate" OR death OR mortality OR "mortality rate" OR "mortality risk" OR prognosis OR "progression") AND DOCUMENT TYPES: (ARTICLE) AND RESEARCH AREAS: (ONCOLOGY OR GENERAL INTERNAL MEDICINE OR GASTROENTEROLOGY HEPATOLOGY OR IMMUNOLOGY OR LIFE SCIENCES BIOMEDICINE OTHER TOPICS OR SURGERY OR NUTRITION DIETETICS OR PUBLIC ENVIRONMENTAL OCCUPATIONAL HEALTH OR PATHOLOGY OR GERIATRICS GERONTOLOGY OR PHARMACOLOGY PHARMACY) |

**Material S1.** Reparameterization calculations for odds ratios and hazard ratios from included articles.

Attached as a separate .pdf file. (Supplementary material 2)

**
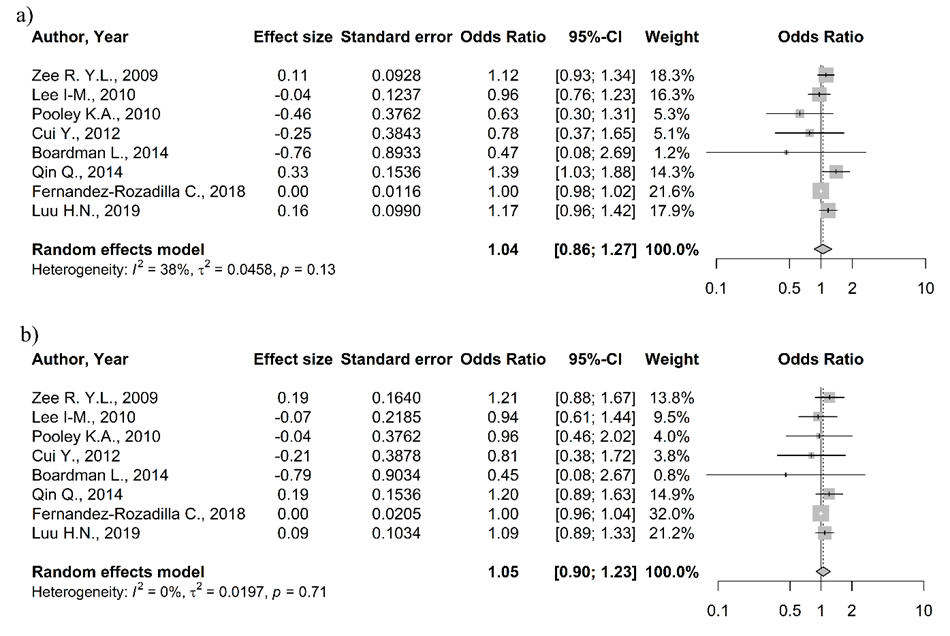
**

**Figure S1.** Forest plot summarizing the association between telomere length in peripheral blood leukocytes and risk of colorectal cancer using random effects model for quartiles of telomere length a) quartile 2 vs quartile 1; and b) quartile 3 vs quartile 1. (References: Zee RY 2009 [32], Lee IM 2010 [31], Pooley KA 2010 [33], Cui Y 2012 [34], Boardman L 2014 [29], Qin Q 2014 [20], Fernandez-Rozadilla C 2018 [30], Luu HN 2019 [21])


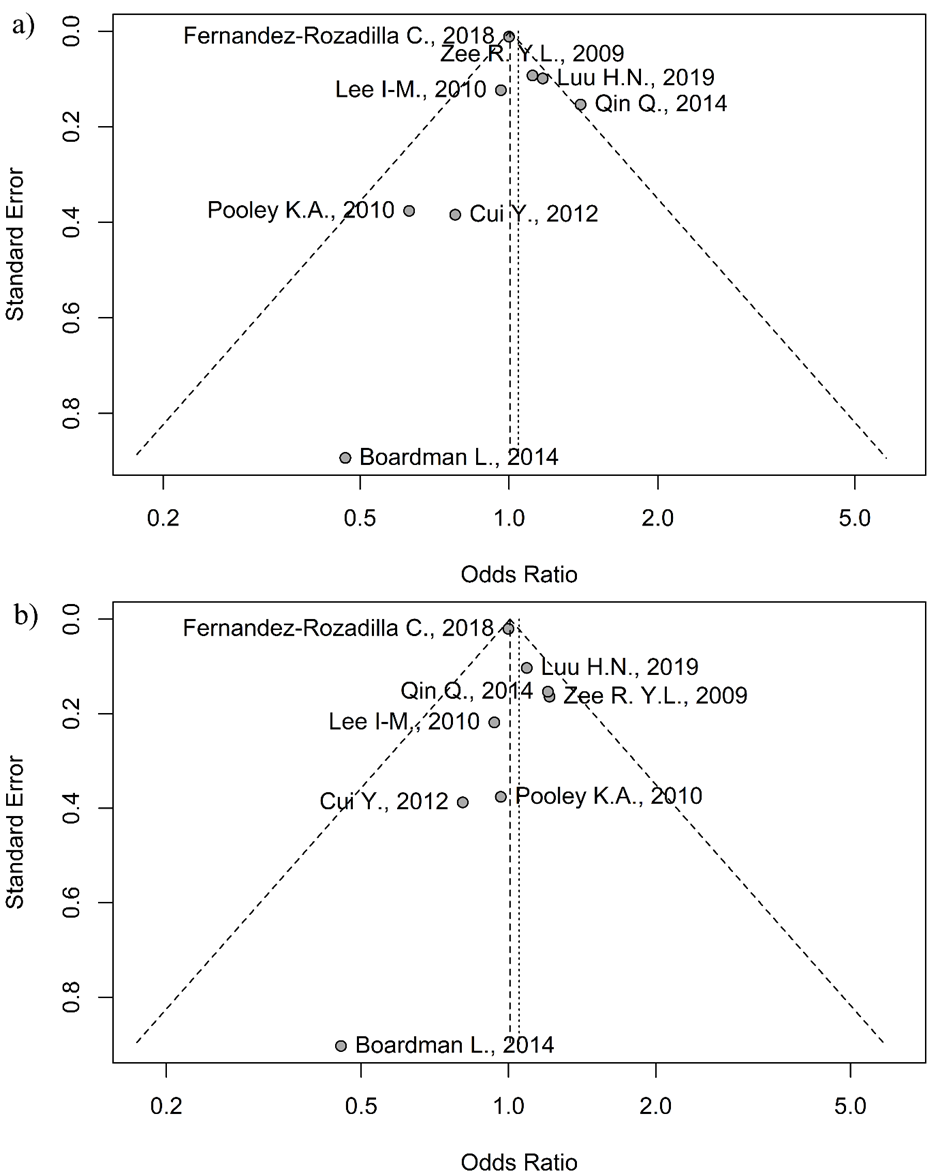


**Figure S2.** Funnel plots assessing potential publication bias of included studies in meta-analysis of telomere length and colorectal cancer risk for quartiles of telomere length a) quartile 2 vs quartile 1; and b) quartile 3 vs quartile 1. (References: Zee RY 2009 [32], Lee IM 2010 [31], Pooley KA 2010 [33], Cui Y 2012 [34], Boardman L 2014 [29], Qin Q 2014 [20], Fernandez-Rozadilla C 2018 [30], Luu HN 2019 [21])

**Table S2.** Comparison of our reparametrized odds ratios for the association between telomere length and colorectal cancer risk with three other published meta-analyses (Naing *et. al,* 2017 [18]; Zhu *et. al,* 2016 [16]; Zhang *et. al,* 2017 [41]).

| **Author, Year** | **TL^1^ categorization** | **Reported OR (95% CI)** | **Calculated OR (95% CI)** | | | |
| --- | --- | --- | --- | --- | --- | --- |
|  |  |  | **Our analysis** | **Naing et al., 2017^2^** | **Zhu et al., 2016^2^** | **Zhang et al., 2017** |
| Zee R.Y.L., 2009 [32] | Continuous | 1.25 (0.86-1.81) | 1.35 (0.82-2.24) | 1.25 (0.86-1.82) | Not included | Not included |
| Lee I-M., 2010 [31] | Continuous | 0.94 (0.65-1.38) | 0.90 (0.46-1.76) | 0.94 (0.73-1.54) | Not included | 1.08 (0.65-1.81) |
| Pooley K.A., 2010 [33] | TL Q4 (shortest)/ Q1 (longest) | 1.13 (0.54-2.36) | 0.89 (0.42-1.85) | 0.89 (0.42-1.85) | Not included | 0.90 (0.48-1.70) |
| Cui Y., 2012 [34] | TL Q1 (shortest)/ Q3  TL Q5 (longest)/ Q3 | 1.56 (0.92-2.64)  1.61(0.94-2.75) | 1.04 (0.38-2.88) | 0.64 (0.38-1.09) | 0.83 (0.65-1.06) | Not included |
| Boardman L., 2014 [29] | *Age >50*  P5 (shorter)/ P50  P95 (longer)/ P50 | 3.53 (1.35-9.25)  1.36 (0.61-3.05) | 0.56 (0.06-5.32) | 0.28 (0.11-0.75) | Not included | Not included |
| Qin Q., 2014 [20] | TL shorter/ longer | 1.47 (1.09-1.99) | 0.68 (0.50-0.92) | 0.68 (0.50-0.92) | 0.78 (0.64-0.94) | Not included |

^1^TL telomere length; ^2^ORs displayed as reciprocal for comparison.
