# Supplementary material for: Association of Telomere Length with Colorectal Cancer Risk and Prognosis: A Systematic Review and Meta-Analysis
Source: Cancers (Basel). 2023 Feb 11;15(4):1159. doi: 10.3390/cancers15041159 (PMC9954736; doi:10.3390/cancers15041159)
Supplement: Supplementary file 1 [file cancers-15-01159-s001.zip › cancers-2140088-supplementary - XML/Supplementary_material2_FINAL.pdf]

Supplementary Material: Calculation Details

Meta-Analysis Calculations: Risk

The papers we included in this meta-analysis estimated different types of associations. Some kept telomere length (TL) as a continuous variable (after log-transformation); others divided it into quartiles (Q) or quintiles (qui). Some papers considered higher telomere length as the exposure, while others considered the reverse. In this document, we demonstrate how we converted the estimates from each paper to estimates we could combine. For the papers examining risk, we decided to estimate the odds ratio (OR) for Q4 vs. Q1, with case-control status of colorectal cancer (CRC) as the outcome. We also estimate ORs for Q3 and Q2 vs. Q1 using similar arguments, but we do not make those calculations explicit here. Throughout, we rely on the following assumptions:

- For papers that quote an OR for continuous log-transformed T/S ratios: We assume the distribution of log-transformed T/S ratios is approximately normal, and we assume the linearity assumption in the model is correct. For these papers, we note whether there is evidence in the paper that the normality assumption is reasonable.
- For papers that quote an OR for a quantile-based division other than quartiles: We assume the OR is approximately constant within the quantile-based division provided.

We also use the facts that $\text{S.D.}(X \pm Y) \leq \text{S.D.}(X) + \text{S.D.}(Y)$ and $\text{S.D.}(aX) = |a|\text{S.D.}(X)$. Further details are provided as needed about each paper in what follows.

1. Zee et al. (2009)

In this paper, they describe the distribution of T/S ratios as having a skewed distribution, and they therefore log-transformed the data, though they do not say explicitly whether the transformed data was approximately normal. They estimated the OR between the log-transformed variable and risk of CRC. Their results are not statistically significant, but the direction of the association is consistent with longer telomere length being associated with increased odds of CRC. The relevant point estimate is:

$$\text{paper OR} = 1.249, 95\% \text{ CI} = (0.863, 1.808).$$

Here, we describe in detail how we converted this estimate to an estimate of Q4 vs. Q1. Let X be the log-transformed T/S ratio variables. Based on the sample means among cases and controls, the overall sample mean of X is 3.518 and the overall sample standard deviation is 0.592. In a standard normal distribution the median of Q1 is -1.150 and the median of Q4 is 1.150. Therefore, the Q4 vs. Q1 difference is 2.301. Thus, if β_1 is the log-OR for a one unit increase in X, then $2.301 \cdot \text{SD}_X \cdot \beta_1 = 1.361\beta_1$ is the log-OR for an increase of 2.301 standard deviations of X, and so, our estimate for the OR for Q4 vs. Q1 is:

$$\text{desired OR} = 1.353, 95\% \text{ CI} = (0.818, 2.239).$$

2. Lee et al. (2010)

In this paper, they describe the distribution of T/S ratios as having a skewed distribution, and they therefore log-transformed the data; however, they do not say explicitly whether the transformed data was approximately normal. They estimated the OR between the log-transformed variable and risk of CRC. Their results are not statistically significant, but the direction of the association is consistent with longer telomere length being associated with reduced odds of CRC. The relevant point estimate is:

$$\text{paper OR} = 0.943, 95\% \text{ CI} = (0.647, 1.376).$$

Our approach for this paper was identical to that described for Zee et al. (2009). Based on the sample means among cases and controls, the overall sample mean of X is 4.505 and the overall sample standard deviation is 0.773. If β_1 is the log-OR for a one unit increase in X, then $2.301 \cdot \mathrm{SD}_X \cdot \beta_1 = 1.778\beta_1$ is the log-OR for an increase of 2.301 standard deviations of X, and so, our estimate of the OR for Q4 vs. Q1 is:

$$\text{desired OR} = 0.901, 95\% \text{ CI} = (0.461, 1.762).$$

3. Pooley et al. (2010)

In this paper, the estimates are given for quartiles, but their quartiles are labeled in the opposite way, so that Q1 represents the group with highest telomere length and Q4 the lowest. They estimate Q4 vs. Q1 using their definitions – i.e., Q1 vs. Q4 using our definitions – so we flip the association. We compared two approaches:

- inverting the OR and confidence interval
- calculating the OR and confidence interval assuming the standard error for the log-OR estimate was the maximum standard error of the log-OR estimates for Q1, Q2, and Q3 vs. Q4.

The results were nearly identical; the results for the second approach are shown. The reported OR is:

$$\text{paper OR} = 1.13, 95\% \text{ CI} = (0.54, 2.36).$$

The desired OR is:

$$\text{desired OR} = 0.885, 95\% \text{ CI} = (0.423, 1.850).$$

4. Cui et al. (2012)

In this study, they reported on ORs comparing quintiles, and used the third quintile (qui3) as the reference group. The reported association was U-shaped. To convert to quartiles, we took linear combinations. For example, we assume the (log-OR) effect of Q1 vs. qui3 is 4/5 the effect of qui1 vs. qui3 plus 1/5 the effect of qui2 vs. qui3. We then estimated the effect of Q4 vs. Q1 by decomposing the desired OR as:

$$\frac{\text{odds(Q4)}}{\text{odds(Q1)}} = \frac{\text{odds(Q4)}}{\text{odds(qui3)}} \cdot \frac{\text{odds(qui3)}}{\text{odds(Q1)}}$$

i.e.,

$$\text{log-OR}_{\text{Q4 vs. Q1}} = \text{log-OR}_{\text{Q4 vs. qui3}} - \text{log-OR}_{\text{qui3 vs. Q1}} = \text{log-OR}_{\text{Q4 vs. qui3}} + \text{log-OR}_{\text{Q1 vs. qui3}}$$

The ORs from the paper that we relied on were:

quintile (reference qui3)	OR	CI: lower bound	CI: upper bound
qui1	1.56	0.92	2.64
qui2	1.30	0.86	1.97
qui4	1.38	0.90	2.11
qui5	1.61	0.94	2.75

And we estimated the OR for Q4 vs. Q1 as:

$$\text{desired OR} = 1.038, 95\% \text{ CI} = (0.375, 2.876).$$

The conversion from the effect estimates from quintiles (reference qui3) to quartiles (reference Q1) is illustrated in the graph below.

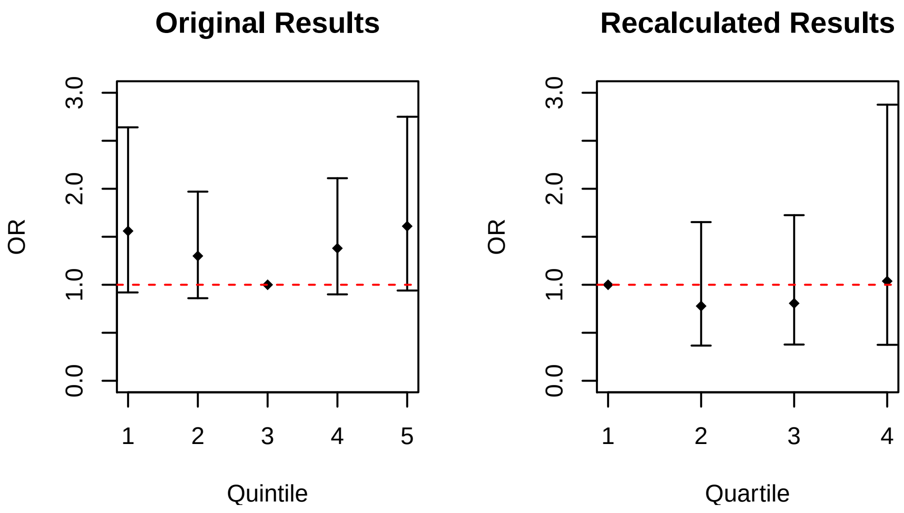

5. Boardman et al. (2014)

In this paper, they give estimates for many percentiles (e.g., 99th percentile, 95th percentile) vs. the 50th percentile. To use these to get the estimates we want, we assume that the the estimate for the 99th percentile is approximately constant for individuals from the 100th percentile to the 97th percentile, where 97 is midway between 99 and the next listed value, 95. We assume the estimate for the 95th percentile is approximately constant for individuals from the 97th to the 92.5th, where 92.5 is midway between 95 and the next listed value, 90. (And so on.) After making those assumptions, the calculation proceeds as in Cui et al. (2012), where we take linear combinations of estimates weighted according the amount of the underlying distribution they should cover in order to convert these percentile estimates to an estimate of Q4 vs. Q1. Also, note that for this paper we use the age > 50 results. We do not here list out all of the odds ratios we used in this calculation; they are tabulated in the right panel of Table 3 of the original paper. The estimated OR for Q4 vs. Q1 is:

$$\text{desired OR} = 0.555, 95\% \text{ CI} = (0.0579, 5.319).$$

6. Qin et al. (2014)

In this paper, the estimates are given for quartiles, but their reference group is Q4. The paper OR (for Q1 vs. Q4) is:

$$\text{paper OR} = 1.47, 95\% \text{ CI} = (1.09, 1.99).$$

We proceeded as we did in Pooley et al. (2010). The desired OR is:

$$\text{desired OR} = 0.680, 95\% \text{ CI} = (0.503, 0.919).$$

7. Fernandez-Rozadilla et al. (2018)

In this paper, they do not talk much about the distribution of telomere length, but they do keep it as a continuous variable. Their estimate is:

$$\text{paper OR} = 1.00, 95\% \text{ CI } (0.88, 1.14).$$

We proceed as we did for Zee et al. (2009). Our estimate of the OR for Q4 vs. Q1 is:

$$\text{desired OR} = 1.00, 95\% \text{ CI} = (0.939, 1.065).$$

8. Luu et al. (2019)

This paper looked at Q4 vs. Q1, so no conversion was needed. The desired OR is the paper OR, which is:

$$\text{desired OR} = \text{paper OR} = 1.32, 95\% \text{ CI} = (1.08, 1.62).$$

Meta-Analysis Calculations: Survival

For the studies looking at survival, we used similar approaches to those listed above, though our central parameter of interest was different – for survival, we tried to extract the hazard ratio (HR) associated with Q1 vs. Q2-4 – i.e., for the *lowest* quartile of telomere length versus *everyone else*.

1. Chen et al. (2014)

The results of this paper suggest that shorter TL is associated with poorer survival. The main results in the paper look at the upper 40.8 % vs. the lower 59.2 %, but decile results (with the lowest decile as the reference) are available in the supplementary material, and we use those to estimate our desired HR for Q1 vs. Q2-4. Our approach is similar to what we did for Cui et al. (2012) and assumes that the estimated HRs are approximately constant across each decile.

Typically, we would expect the confidence interval on the log scale to be symmetric about the log-HR point estimate. This did not quite hold true for all of the results in this paper, but in such cases, we took the length from the lower confidence bound to the point estimate and the length from the point estimate to the upper confidence bound (on the log scale) and averaged these to get the half-width of the confidence interval.

The resulting HR of interest is:

$$\text{desired HR} = 3.149, 95\% \text{ CI} = (1.850, 5.361).$$

2. Svenson et al. (2016)

This paper found a favorable relationship between shorter telomere length and survival. They estimate Q1 vs. Q2-4, so we do not need to alter their estimate. The (fully adjusted) HR is:

$$\text{desired HR} = \text{paper HR} = 0.52, 95\% \text{ CI} = (0.15, 1.76).$$

3. Gertler et al. (2004)

In this paper, they find that a higher value of the ratio between telomere lengths in cancer and noncancer tissue was associated with poorer survival, when the ratio was dichotomized at 0.90. 14 (25%) patients had ratio above 0.90 and 43 (75%) below. They say that CART was used to find the cutpoint of 0.90, and they do not discuss the shape of the distribution of the ratio variable. We therefore proceeded as we did in, e.g., Cui et al. (2012) and assumed the HR was approximately constant comparing someone in the upper quartile to someone in the lower three quartiles, and reconstructed an HR comparing someone in the lower quartile to the upper three quartiles; the relationship would be expected to attenuate in this conversion. The HR provided in the paper, for the higher TL group based on their cutpoint, is:

$$\text{paper HR} = 3.3, 95\% \text{ CI} = (1.2, 9.0).$$

The HR we use for Q1 vs. Q2-4 is:

$$\text{desired HR} = 0.746, 95\% \text{ CI} = (0.582, 0.955).$$

4. Valls et al. (2010)

In this paper, they also look at the ratio between telomere lengths in cancer and noncancer tissue, and also find that a higher value of this ratio is associated with poorer survival. They also use CART to find the cutpoint of 1 for the ratio, and they do not discuss the shape of the distribution of the ratio variable. They compare 29 (23.2%) patients with ratio above 1 to 96 (76.8%) below. The HR provided in the paper, for the higher TL group, is:

$$\text{paper HR} = 2.442, 95\% \text{ CI} = (1.198, 4.976).$$

The HR we use for Q1 vs. Q2-4 is:

$$\text{desired HR} = 0.813, 95\% \text{ CI} = (0.689, 0.959).$$

5. Suraweera et al. (2016)

In this paper, they report a null relationship between cRTL/nRTL ratio (modeled as a continuous variable) and OS; the relevant HR is:

$$\text{paper HR} = 0.99, 95\% \text{ CI} = (0.75, 1.32).$$

To convert the result from a linear effect to a Q1 vs. Q2-4 effect, we assume that the distribution of the ratio is normal, and then proceed analogously to what we did for Zee et al. (2009). We need the sample standard deviation of the cRTL/nRTL telomere length measure, which did not appear to be calculated directly. We therefore calculated it by combining the sample standard deviations listed in Table 2 separated by MSI status. The HR we estimate for Q1 vs. Q2-4 is:

$$\text{desired HR} = 1.014, 95\% \text{ CI} = (0.678, 1.519).$$
